# Enhanced sugar accumulation and regulated plant hormone signalling genes contribute to cold tolerance in hypoploid *Saccharum spontaneum*

**DOI:** 10.1186/s12864-020-06917-z

**Published:** 2020-07-22

**Authors:** Hongli Yang, Tianju Wang, Xinghua Yu, Yang Yang, Chunfang Wang, Qinghui Yang, Xianhong Wang

**Affiliations:** 1 Sugarcane Research Institute, Yunnan Agricultural University, Kunming, 650201 Yunnan Province PR China; 2 Chuxiong normal university, Chuxiong, 675000 Yunnan Province PR China; 3 Wenshan Academy of Agricultural Sciences, Wenshan, 663000 Yunnan Province PR China

**Keywords:** Sugarcane, *Saccharum spontaneum*, Hypoploidy, Cold tolerance, RNA-seq, Sugar accumulation, Hormonal change

## Abstract

**Background:**

Wild sugarcane *Saccharum spontaneum* plants vary in ploidy, which complicates the utilization of its germplasm in sugarcane breeding. Investigations on cold tolerance in relation to different ploidies in *S. spontaneum* may promote the exploitation of its germplasm and accelerate the improvement of sugarcane varieties.

**Results:**

A hypoploid clone 12–23 (2n = 54) and hyperploid clone 15–28 (2n = 92) of *S. spontaneum* were analysed under cold stress from morphological, physiological, and transcriptomic perspectives. Compared with clone 15–28, clone 12–23 plants had lower plant height, leaf length, internode length, stem diameter, and leaf width; depressed stomata and prominent bristles and papillae; and thick leaves with higher bulliform cell groups and thicker adaxial epidermis. Compared with clone 15–28, clone 12–23 showed significantly lower electrical conductivity, significantly higher water content, soluble protein content, and superoxide dismutase activity, and significantly higher soluble sugar content and peroxidase activity. Under cold stress, the number of upregulated genes and downregulated genes of clone 12–23 was higher than clone 15–28, and many stress response genes and pathways were affected and enriched to varying degrees, particularly sugar and starch metabolic pathways and plant hormone signalling pathways. Under cold stress, the activity of 6-phosphate glucose trehalose synthase, trehalose phosphate phosphatase, and brassinosteroid-signalling kinase and the content of trehalose and brassinosteroids of clone 12–23 increased.

**Conclusions:**

Compared with hyperploid clone 15–28, hypoploid clone 12–23 maintained a more robust osmotic adjustment system through sugar accumulation and hormonal regulation, which resulted in stronger cold tolerance.

## Background

Sugarcane (*Saccharum* spp.) is an important sugar and energy crop, contributing 80% of the world’s sugar production and 40% of that of ethanol [1]**.** The high sugar content genes of modern sugarcane varieties derived from tropical species (*S. officinarum*), however, are poorly resistant to biotic and abiotic stresses [2]**.***S. spontaneum* belongs to the perennial herb of the family Gramineae and genus *Saccharum,* has both asexual and sexual reproductive abilities [3, 4], can grow in a variety of environments, such as drought, cold and high salt conditions [5], has varied phenotypes and strong adaptability, can provide rich genetic diversity for sugarcane breeding, and is the main source of desirable genes resistant to pests, diseases, cold, and drought [6, 7].

Polyploidization is a major driver of speciation [8]. Polyploidy is the characteristic of most important crops [9], such as bamboo [8], rice [10], citrus [11], wheat [11], canola [12], cotton [13] and potato [13]. According to reported estimates, 30–35% of known species and nearly 75% of Gramineae plants are polyploids [14]. Polyploids have a wider range of tolerability profiles. *S. spontaneum* has a basic chromosome number x = 8 with a ploidy level of 5 to 16 [15], which is a typical polyploid plant, including euploids or aneuploids with chromosome numbers ranging from 2n = 40 to 128 [16]. Moreover, polyploid *S. spontaneum* is capable of adapting to different environments [17], indicating that this species can be used in sugarcane breeding. Nevertheless, only the *S. spontaneum* lines Glagah (2n = 112), Indian (2n = 64), and Yacheng (2n = 64, 80) have been successfully exploited to date [18, 19].

Low temperature is the most important environmental factor limiting the productivity and geographical distribution of plants around the world [20]. Climate change exacerbates the adverse effects of low temperature stress and leads to an increase in the frequency of extreme weather [21]. Sugarcane originated in the tropics. With increasing consumer demand, its planting belt has gradually expanded to the subtropical region [22]. However, subtropical winter has adversely affected the cultivation of sugarcane. The response of different sugarcane varieties significantly differs under cold stress [23] and involves differentially expressed genes [5], and miRNAs also play an important role in the cold tolerance of sugarcane [24]. Under cold stress, numerous upregulated genes were identified in *S. spontaneum* [25], and carbohydrate metabolism was determined to be the most significantly enriched functional pathway [26]. In-depth studies on the cold tolerance of *S. spontaneum* at the transcriptomic level are thus essential to its improved cold tolerance in this economically significant sugarcane species.

The Sugarcane Research Institute of Yunnan Agricultural University located in Kunming city, Yunnan Province, China has collected and preserved nearly 600 clones of *S. spontaneum* germplasm resources since 1985 and has identified 10 types of ploidies, namely, 2n = 40, 48, 54, 60, 64, 78, 80, 88, 92, and 96 [27]. Physiological and biochemical analyses have shown that the tolerance of different ploidies of *S. spontaneum* to low temperature varied, with the hypoploid clone 12–23 (2n = 54) being highly cold-resistant, whereas the hyperploid clone 15–28 (2n = 92) was typically cold-sensitive (Additional file 1: Supplement). The two clones were used in this study to explore the cold resistance of different ploidies of *S. spontaneum* in terms of morphological, physiological, and molecular characteristics, which may guide sugarcane breeding in generating new varieties with improved cold resistance.

## Results

### Morphological and microscopic comparison of two clones

**Fig. 1:**
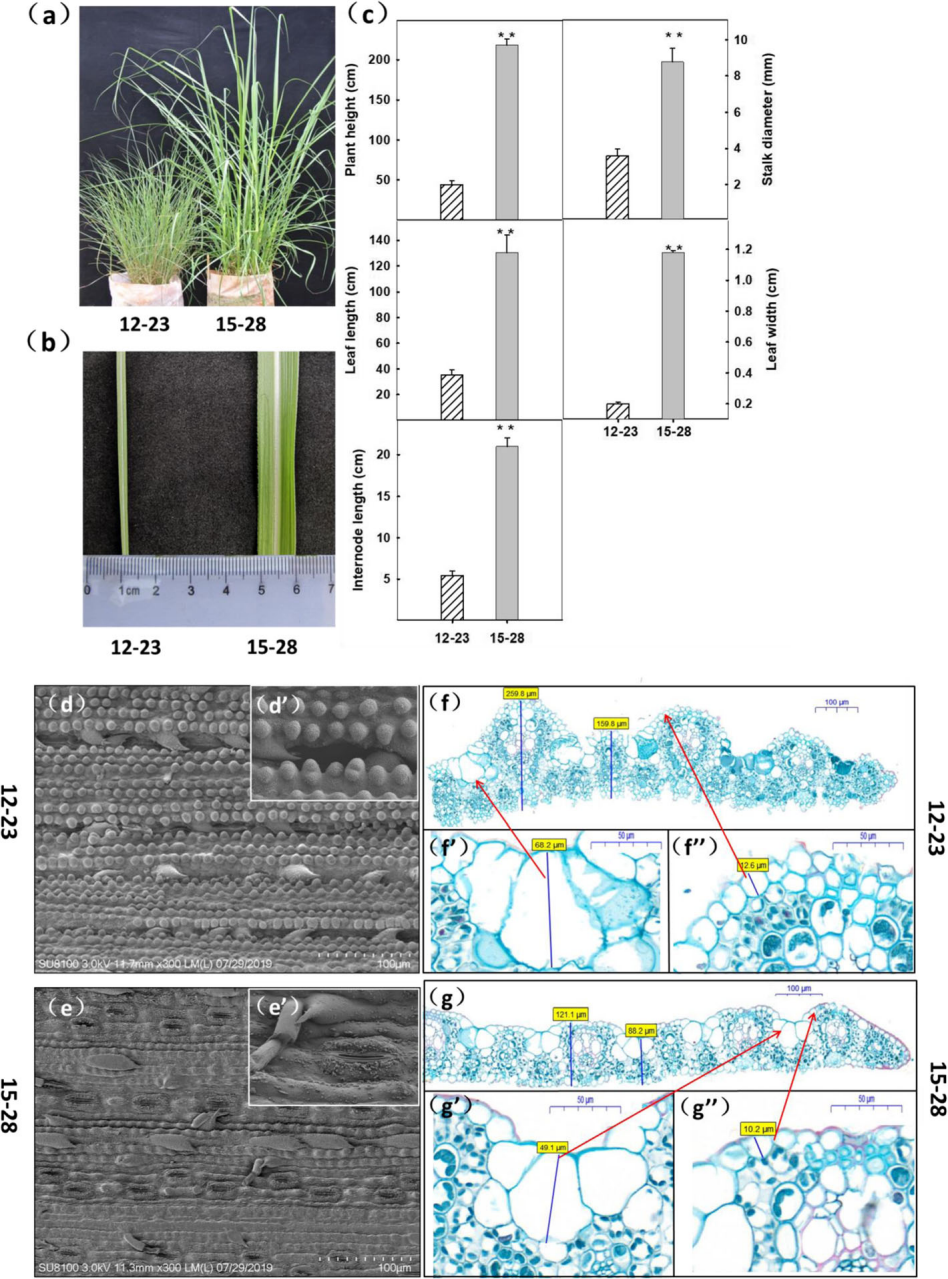
Morphological features of the *Saccharum spontaneum* clones 12–23 and 15–28. **a** Plants cultivated for four months. **b** Leaves of plants cultivated for four months. **c** Plant height, leaf length, internode length, stem diameter, and leaf width of plants at the maturity stage (** indicates *p* < 0.01). Scanning electron micrograph of stomata of (**d**) (**d**’) clone 12–23 and (**e**) (**e**’) clone 15–28. Thickness of leaf blade, bulliform cells, and adaxial epidermis of (**f**) (**f**’) (**f**”) clone 12–23 and (**g**) (**g**’) (**g**”) clone 15–28

The morphological characteristics of clones 12–23 and 15–28 showed significant differences under the growth conditions of the greenhouse. Compared with clone 15–28 plants, clone 12–23 plants were relatively shorter (Fig. 1a), and leaf blades were narrower (Fig. 1b). The morphological characteristics of the two clones were further measured at the maturity stage. The five morphological characteristics, including plant height, leaf length, internode length, stem diameter, and leaf width, of clone 12–23 were smaller than those of clone 15–28 (Fig. 1c). In addition, leaf anatomical analysis showed that the stomata of the 12–23 clone were depressed, and the bristles and papillae were prominent (Fig. 1d and d’), whereas the stomata of clone 15–28 were not depressed, and their bristles and papillae were fewer in number and not prominent (Fig. 1e and e’). The comparison of transverse sections of the leaves of the two clones showed that the leaf thickness of clone 12–23 was considerably larger than that of clone 15–28 (Fig. 1f and g); the bulliform cell groups of clone 12–23 was higher than that of the clone 15–28 (Fig. 1f’ and g’); and the adaxial epidermal thickness of clone 12–23 is greater than that of clone 15–28 (Fig. 1f” and g”).

### Difference in physiological indexes between the two clones after cold stress

**Fig. 2:**
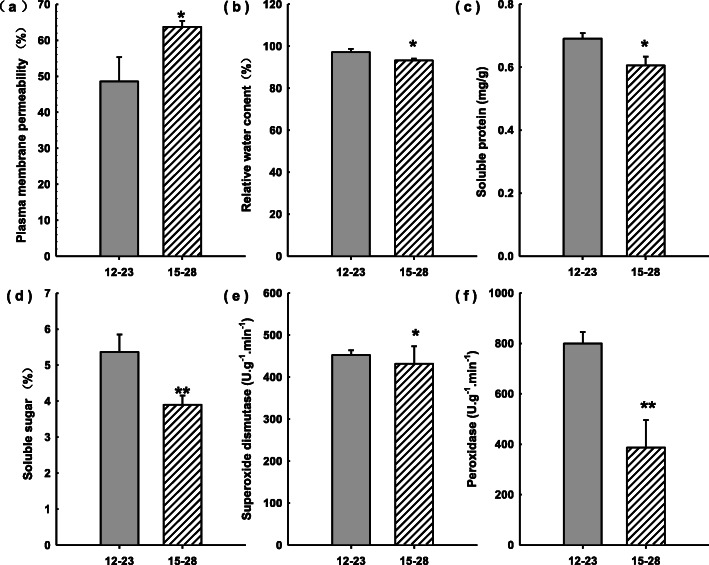
Differences in various physiological characteristics between clones 12–23 and 15–28 after low temperature stress. **a** Cell membrane permeability. **b** Relative water content of the leaves. **c** Soluble protein content. **d** Soluble sugar content. **e** Superoxide dismutase content. **f** Peroxidase content. (* indicates *p* < 0.05 ** indicates *p* < 0.01)

After cold stress, the physiological indexes, including plasma permeability, leaf water content, soluble protein content, soluble sugar content, superoxide dismutase activity, and peroxidase activity were significantly different between clones 12–3 and 15–28 (Fig. 2). The electrical conductivity of clone 12–23 was significantly lower than that of clone 15–28 (Fig. 2a). The relative water content, soluble sugar content, soluble protein content, superoxide dismutase activity, and peroxidase activity of clone 12–23 were higher than those of clone 15–28, of which the difference in relative water content, soluble protein content, and superoxide activity between clones reached significant levels (*P* < 0.05), and the difference in soluble sugar content and peroxidase activity between clones was also significant (*P* < 0.01) (Figs. 2b–f).

### Differences in Transcriptomic data of clones 12–23 and 15–28 after cold stress

Transcription is the first step in gene expression, and it is also a key step in expression regulation. Therefore, transcriptome data analysis of the two ploidy *S. spontaneum* was performed to further understand the cold tolerance mechanism of the hypoploid clone 12–23 at the molecular level. Transcriptome data of the clone 12–23 low temperature stress treatment group (LL), clone 15–28 low temperature stress treatment group (HL), clone 12–23 control group (LC) and clone 15–28 control group (HC) were compared. After HiSeq2500 high-throughput sequencing, clean reads were obtained for the four libraries (LL, LC, HL, and HC): 55300316, 60,412,261, 58,497,202, and 45,591,750 (Additional file 2: Table S1). The data were used in the database for NR (non-redundant protein sequences), Swiss-Prot (a manually annotated and reviewed protein sequence database), Pfam (Protein family), KOG (Eukaryotic Orthologous Groups) (e-value< 0.00001) comparison, and 86,275 genes were annotated. Among these genes, the most annotated genes in the Nt database were 75,297 (66.41%) (Additional file 3: Table S2).

**Fig. 3:**
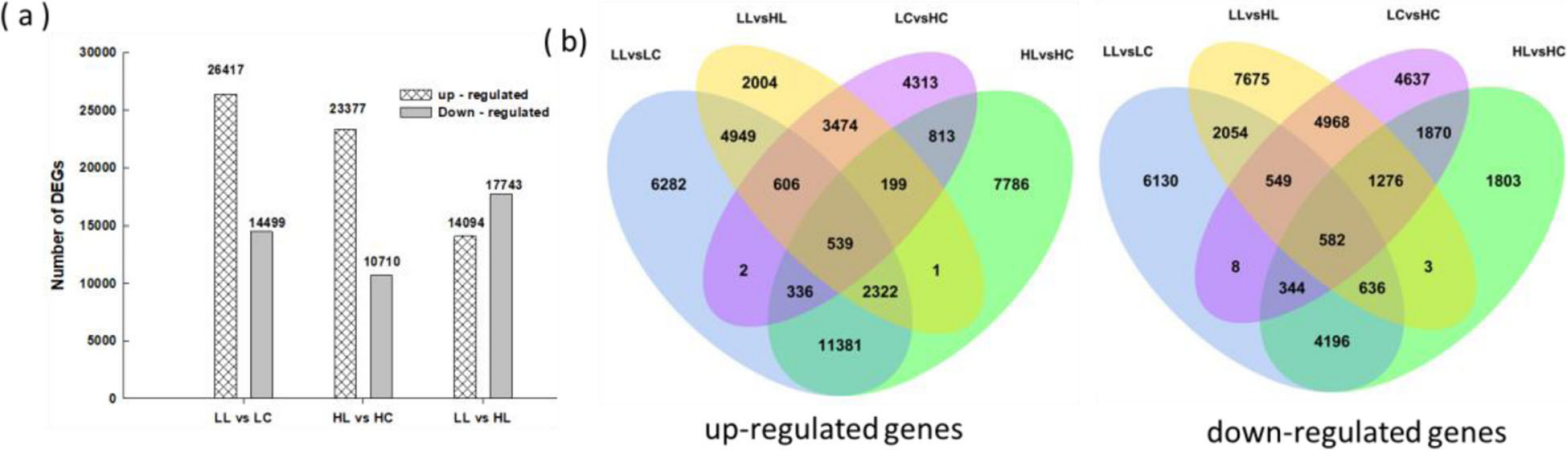
Differentially expressed genes (DEGs) in clones 12–23 and 15–28 under control and cold conditions. **a** Number of upregulated and downregulated genes in clones 12–23 and 15–28 exposed to cold vs. control conditions (LL vs. LC, HL vs. HC) and in clone 12–23 vs. 15–28 under cold conditionss (LL vs HL) as revealed by RNA-seq. **b** Venn diagram analysis of the common and specific upregulated and downregulated genes using different pairwise comparisons

Transcriptome data of clones 12–23 included the low temperature stress group (LL) and control group (LC), and transcriptome data of clones 15–28 included the low temperature stress group (HL) and control group (HC). The transcriptomic data of clones 12–23 and 15–28 were analysed in four pairwise comparisons: LL vs. HL, LC vs. HC, LL vs. LC and HL vs. HC. The total number of differentially expressed genes (DEGs) in LL vs. LC was 40,916, of which 26,417 genes were upregulated and 14,499 genes were downregulated. The total number of DEGs in HL vs. HC was 34,087, of which 23,377 genes were upregulated and 10,710 genes were downregulated, indicating that the cold-tolerant clone 12–23 had more up- and downregulated genes than the cold-sensitive clone 15–28. In the LL vs. HL comparison, the total number of DEGs was 31,837, of which 14,094 genes were upregulated and 17,743 were downregulated (Fig. 3a). Further analysis showed that among the commonly shared DEGs in the four pairwise comparisons, 539 genes were upregulated, and 582 genes were downregulated (Fig. 3b).

**Fig. 4:**
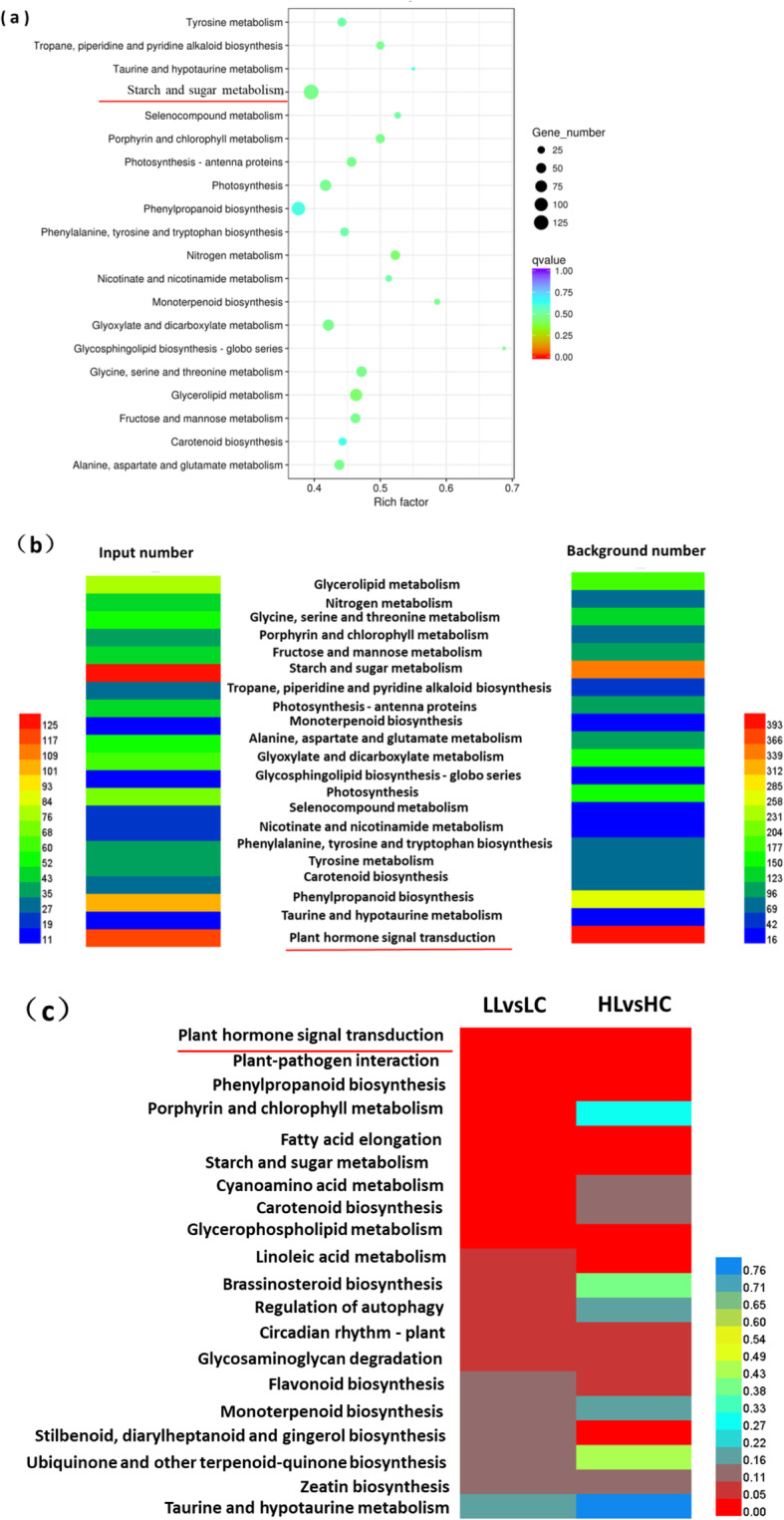
Kyoto Encyclopedia of Genes and Genomes (KEGG) pathway analysis of differentially expressed genes (DEGs). **a** The top 20 enriched pathways of the DEGs between clones 12–23 and clone 15–28 under cold conditions (LL vs. HL). The X-axis indicates the enrichment factor on a scale from 0 to 0.7. The dot colour and size indicate the q-value and gene number as shown on the right. **b** Heat map of input number and background number of the KEGG enriched pathways of the DEGs of clones under cold conditions (LL vs. HL). The left colour panels display the input number of each term from 11 to 125, and the right colour panels display the background number of each term from 16 to 393. **c** Heat map of the KEGG enriched pathways of the DEGs of the pairwise comparisons LL vs. LC and HL vs. HC. Colour panels display the *P*-values of each term from 0 to 0.76

The DEGs identified from the LL vs. HL pairwise comparison were used in Kyoto Encyclopedia of Genes and Genomes (KEGG) pathway enrichment analysis, and 20 major pathways were obtained, of which, the DEGs were primarily enriched in three pathways, namely, starch and sugar metabolism, phenylpropanoid biosynthesis, and glycolysis/gluconeogenesis, and the most significant pathways were the sugar and starch metabolic pathways (Fig. 4a). Moreover, among the pathways obtained through KEGG enrichment analysis of the DEGs identified from LL vs. HL, the plant hormone signal transduction pathway had the highest number of DEGs (Fig. 4b). Further comparison of the *P*-value of the pathways obtained through KEGG enrichment analysis of DEGs identified from the pairwise comparisons of LL vs. LC and HL vs. HC showed that the plant hormone signalling transduction pathway was the most significantly enriched (Fig. 4c). Based on these results, we focused our analysis on the sugar and starch metabolic and plant hormone signalling pathways.

### Enhanced sugar accumulation contributes to cold tolerance in *S. spontaneum*

**Fig. 5:**
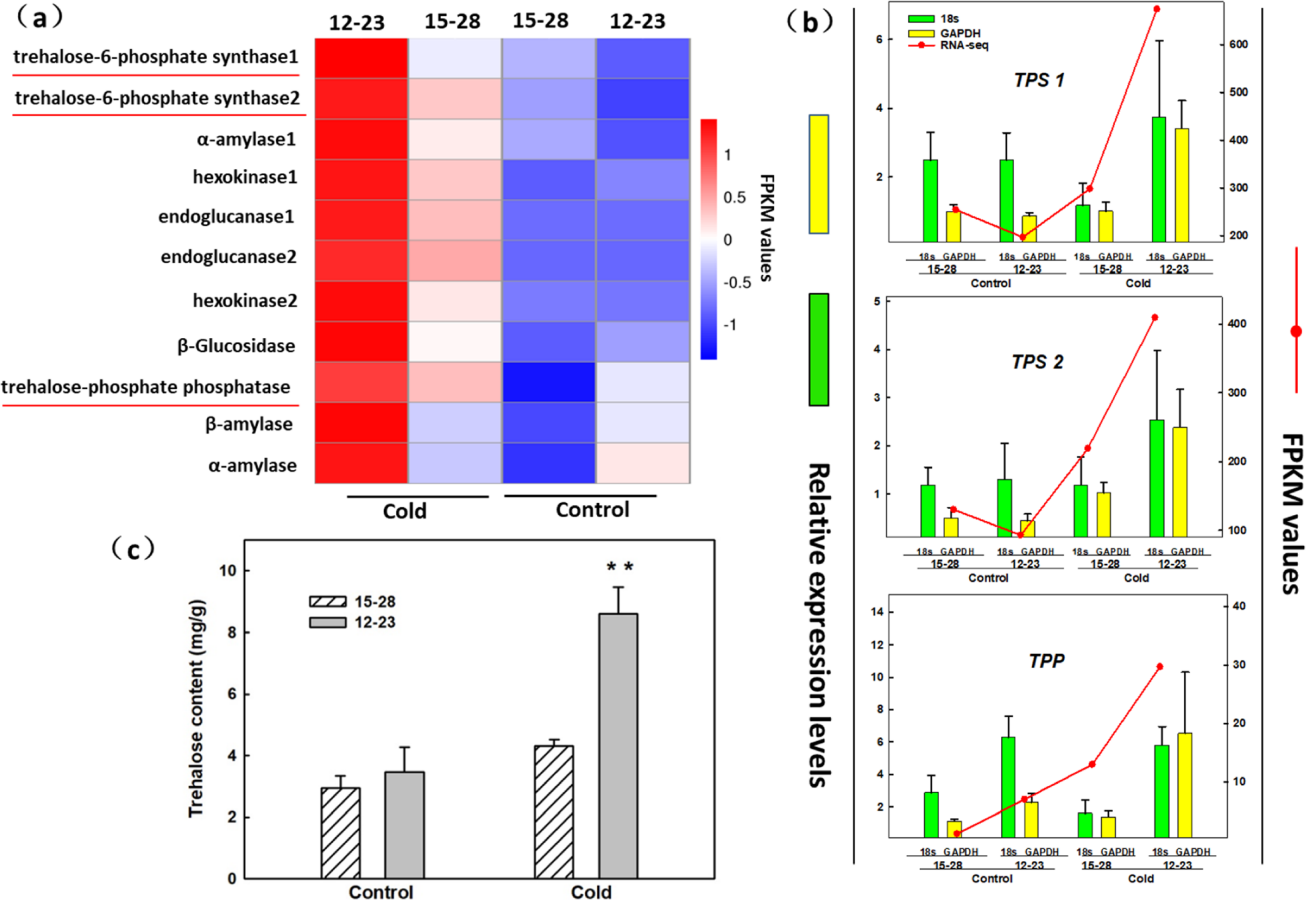
Changes in starch and sugar metabolism, expression levels of TPS and TPP as revealed by RNA-seq and RT-qPCR, and content of trehalose after cold stress. **a** Heat map of the FPKM values generated from the RNA-seq analysis of clones 12–23 and 15–28 under control (column 2 on the right) and cold stress (column 2 on the left) conditions. The IDs of the reference homologous genes and their enzymes collected from the genomic databases are shown on the left. The gradient colour bar code in the upper right corner indicates the normalized FPKM value. **b** Expression levels of TPS1, TPS2, and TPP as revealed by RNA-seq and RT-qPCR. The red curve indicates the FPKM value as revealed by RNA-seq analysis; the green column indicates the value as revealed by RT-qPCR using the 18S rRNA gene as an internal reference; the yellow column indicates the value as revealed by RT-qPCR using the GAPDH gene as an internal reference. **c** Trehalose content of clones 12–23 and 15–28 under control and cold stress conditions (** indicates *p* < 0.01)

KEGG pathway enrichment analysis of DEGs from LL vs. HL showed that the sugar and starch metabolic pathways were most significantly enriched. The DEGs in the sugar and starch metabolic pathways were further analysed by means of ORFfinger alignment and functional annotation (KO and GO), and then the FPKM values of the screened genes were compared. We found that the FPKM values of the 6-phosphate glucose trehalose synthase (*TPS*) and trehalose phosphate phosphatase (*TPP*) genes controlling trehalose synthesis were the highest (Fig. 5a). The differential expression of *TPS1*, *TPS2*, and *TPP* was validated by RT-qPCR using *18S rRNA* and *GAPDH* genes as internal references. The results showed that the differential fold change in the expression of genes detected by RT-qPCR was different from that revealed by RNA-seq, but the trends of gene expression presented by the two methods were largely the same (Fig. 5b) (Additional file 4: Figure S1). Furthermore, we determined the change in trehalose content in clones 12–23 and 15–28 under cold stress and control conditions. Under control conditions, the two clones did not show significant differences in trehalose content. After cold stress, trehalose content increased in both clones, although the trehalose content of clone 12–23 was significantly higher than that of clone 15–28 (Fig. 5c).

### Regulated plant hormone Signalling genes contribute to cold tolerance in *S. spontaneum*

**Fig. 6:**
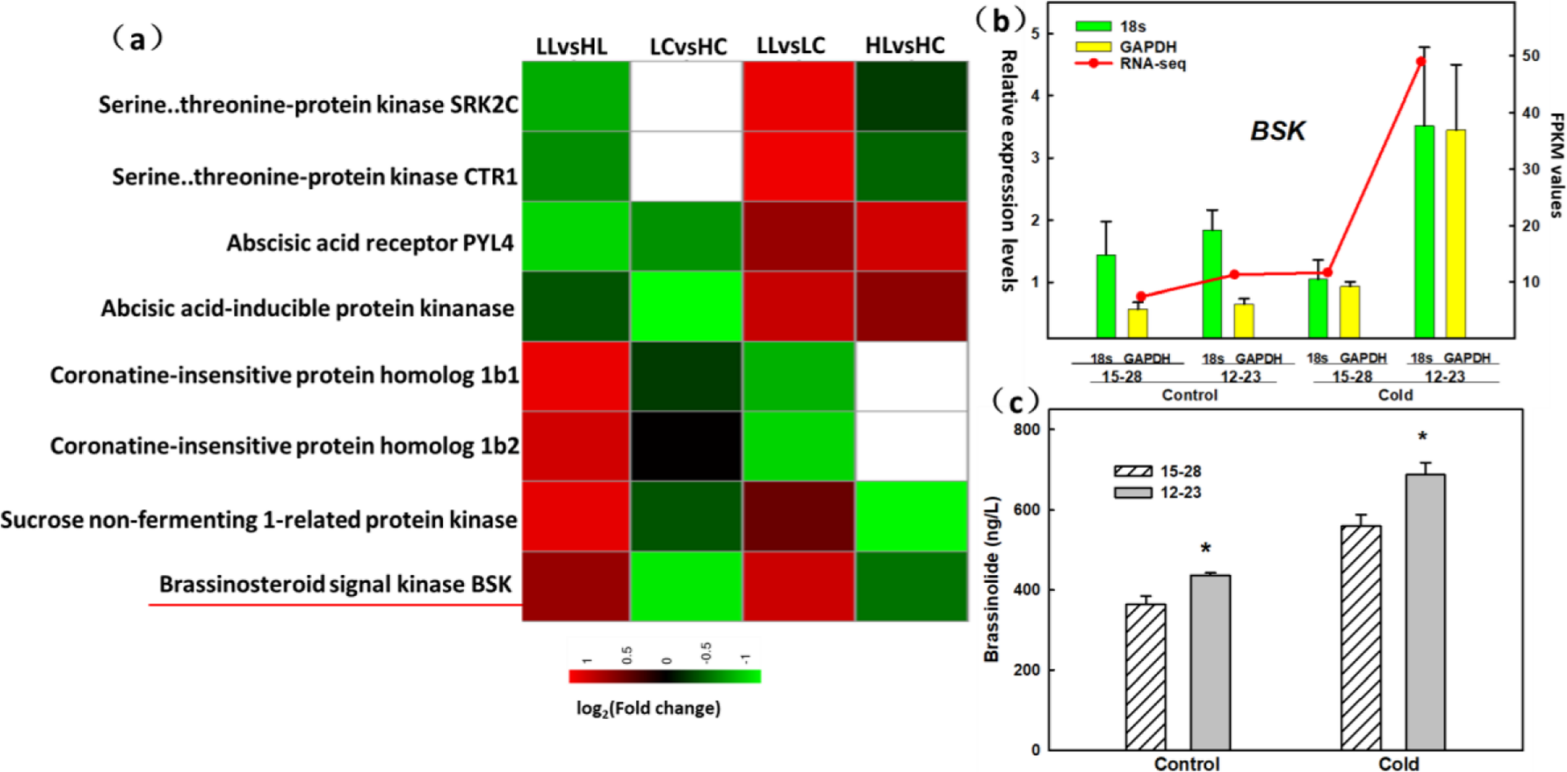
Changes in plant hormone signalling, expression levels of BSK revealed by RNA-seq and RT-qPCR, and brassinosteroid content after exposure to cold stress. **a** Heat map of the log_2_(fold change) value generated by RNA-seq analysis of the four pairwise comparisons (LL vs. HL, LC vs. HC, LL vs. LC, HL vs. HC). The IDs of reference homologous genes and their enzymes collected from the genomic databases are shown on the left. The gradient colour bar code shown below represents the normalized log_2_(fold change) value. **b** Expression levels of BSK as revealed by RNA-seq and RT-qPCR. The red curve indicates the FPKM value generated by RNA-seq; the green column indicates the value generated by RT-qPCR using the 18S rRNA gene as an internal reference; the yellow column indicates the value generated by RT-qPCR using the GAPDH gene as an internal reference. **c** Brassinosteroid content in clones 12–23 and 15–28 plants under control vs. cold stress. (* indicates significant difference, *p* < 0.05)

For the DEGs obtained from RNA-seq analysis, analysis of FPKM value (expression level), log_2_ (fold change), ORFfinger alignment, and functional annotation (KO, GO) revealed that the log_2_ (fold change) value of the brassinosteroid signal kinase (*BSK*) was the highest (Fig. 6a), and for both clones 12–23 and 15–28, the FPKM values increased after cold stress (Fig. 6b). The expression of the *BSK* gene was verified by RT-qPCR using *18S rRNA* and *GAPDH* genes as internal references. The differential fold change in gene expression using RT-qPCR differed from that shown by RNA-seq, but the trend in gene expression presented by the two methods was largely the same (Fig. 6b) (Additional file 5: Figure S2). We further determined the change in brassinosteroid content of clones 12–23 and 15–28 subjected to cold stress. The difference in brassinosteroid content between the two clones was significant under control conditions. After cold treatment, the brassinosteroid content increased in both clones, and its content was significantly higher in clone 12–23 than in clone 15–28 (Fig. 6c).

## Discussion

### Morphological and physiological characteristics of low-ploidy and high-ploidy of *S. spontaneum* vary with cold tolerance

A previous study on *S. spontaneum* indicated that phenotypes differed with ploidy. Low-ploidy plants tend to be short and form thick leaves [28]. Plant leaves are the main organ responding to environmental affecters, and their anatomical features have an important impact on their adaptability to specific environments [29]. As a channel for the leaves to absorb CO_2_ and dissipate moisture, stomata regulate and control the water use efficiency of plants. Stomata are also in the joint point of primary productivity of terrestrial ecosystems with water transpiration and can be used as an indicator of the strength of plant resistance [30]. Depressed stomata may reduce moisture evaporation and are a characteristic of stress-resistant plants [31]. Bulliform cells are unique leaf structures of gramineous plants. Plants with a high number and/or large volume of bulliform cells tend to have greater stress resistance [32]. Water storage and supply of a plant have a close relationship with leaf thickness [33]. The results of this study indicated that the stomata of clone 12–23 were depressed, and the bristles and papillae were prominent, whereas clone 15–28 did not exhibit such characteristics. In addition, clone 12–23 showed taller bulliform cells and thicker adaxial epidermis than that of clone 15–28. Thus, based on the anatomical structure of the leaves, clone 12–23 possesses cold-tolerant characteristics.

When plants are exposed to low temperature, the first change that occurs is cell membrane permeability. The membrane is a protective barrier between plant cells and the external environment. The membrane can not only receive and transmit environmental signals, but also respond to environmental stress. Low temperature may damage cell membranes, thereby increasing their permeability. The relative permeability of the cell plasma membrane is positively correlated with the degree of cell membrane damage [23]; therefore, the value of the membrane permeability is negatively correlated with cold tolerance. Soluble proteins and soluble sugars are important osmoregulation substances in plants. The content of these proteins and sugars increases at low temperature, acting as anti-dehydrating agents to reduce the water potential of cells and enhance the water holding capability, thereby reducing plant damage [34]. Under abiotic stress, plants may accumulate reactive oxygen species (ROS). If not removed, excessive ROS can cause damage to nucleic acids, proteins, and lipids [35]. Plants have evolved an antioxidant defence system, of which superoxide dismutase and peroxidase are the strongest antioxidant enzymes [21] and are important physiological indexes for cold tolerance detection. The results of our study indicated that the physiological indexes of the two clones were significantly different under cold stress. The electrical conductivity of clone 12–23 was lower than that of clone 15–28. The relative water content, soluble sugar content, soluble protein content, superoxide dismutase activity, and peroxidase activity of clone 12–23 were greater than those of clone 15–28. These results indicate that clone 12–23 is more tolerant to cold.

### Transcriptomic difference between cold-resistant and cold-sensitive clones of *S. spontaneum* in response to cold stress

Transcriptional regulation is an important regulatory mechanism of the transcriptome in response to abiotic stress in plants [36]. Studies have shown that cold-tolerant *S. spontaneum* produces more DEGs than other sugarcane varieties under cold stress [5]. In this study, the transcriptomic analysis that clone 12–23 revealed changes in gene expression under cold stress, which might involve more metabolic pathways and regulatory mechanisms to adapt to the cold environment.

Multiple genes interact with each other in different pathways and play roles in the abiotic stress response. KEGG is a database that integrates genomic, chemical, and systemic functional information [37]. Under certain circumstances, soluble sugar and starch can be converted into each other. As an intermediate or end product of metabolism, sugar can regulate many physiological processes such as plant growth, development, and formation of stress resistance, indicating that the sugar and starch metabolic pathways play an important role in the enhancement of cold tolerance in plants [38]. In addition, plant hormones can regulate the growth and development of plants, which include auxins, cytokines, gibberellins, abscisic acid, ethylene, and brassinosteroids. Hormones are involved in the normal development of plants and play an important role in stress responses [39]. In this study, we found that among all the pathways revealed through KEGG enrichment analysis of the DEGs from the LL vs. HL pairwise comparison, the plant hormone signal transduction pathway showed the highest enrichment. Comparison of *P*-values of the KEGG enriched pathways of DEGs from clones 12–23 and 15–28 under cold stress indicated that for the pairwise comparisons of LL vs. LC and HL vs. HC, the plant hormone signalling transduction pathway was the most significantly enriched pathway by KEGG.

### Increase in Trehalose content and changes in plant hormone expression significantly enhances cold tolerance in low-ploidy *S. spontaneum*

Several soluble sugars, such as sucrose, glucose, fructose, ribose, and trehalose, often accumulate under low temperature stress [11]. These sugars can scavenge free radicals and indirectly induce protein synthesis, thereby improving the cold tolerance of plants [25]. The results of this study indicate that the 6-phosphate glucose trehalose synthase (*TPS*) and the trehalose phosphate phosphatase (*TPP*) that synthesize trehalose are significantly different when comparing the two materials (LL vs. HL) under low temperature stress. Trehalose is a safe, nonreducing disaccharide that is found in fungi, bacteria and insects [40]. Trehalose plays a role in protecting biomacromolecules, such as membrane proteins, and hence is significant to organism survival [41]. Under adverse conditions such as drought, cold, and high salt, plants can produce trehalose to help resist these external influences. An earlier study showed that trehalose could be detected only in “resurrected plants” that have notably strong drought tolerance [42]. In 1969, trehalose was detected in sugarcane seedlings [43]. *TPS* and *TPP* are the most important enzymes for the synthesis of trehalose [44]. 6-Phosphate glucose trehalose synthase (*TPS*) catalyses the formation of 6-phosphate trehalose (*T6P*) from UDP-glucose and 6-phosphate glucose, and then *T6P* is catalysed by trehalose phosphate phosphatase (*TPP*) to produce trehalose. *TPS* and *TPP* are also called *OtsA* and *OtsB*, respectively [41]. Studies have shown that tobacco with the trehalose synthesis gene showed strong drought tolerance [45], and the transgenic rice plants overexpressing these genes exhibited significantly enhanced tolerance to cold, drought, and high salt levels [46]. Interestingly, we found that under cold stress, the trehalose content of clone 12–23 was higher than that of clone 15–28, suggesting that *TPS* and *TPP* play an important role in the enhancement of cold tolerance of hypoploid *S. spontaneum.*

Studies have shown that ABA signalling activates the expression of plant cold resistance genes mediated by ABREs (ABA responsive element binding protein) to enhance cold resistance [47]. ABA signal transduction is largely dependent on the protein phosphatase *PP2C* [48]. The results of this study showed that *PP2C* related to ABA synthesis was significantly higher than that of the control group under low temperature stress, indicating that ABA plays an important role in resistance to low temperature stress. The comparison between low-ploidy clones 12–23 and high-ploidy clones 15–28 under low temperature stress indicated that brassinolactone was the major hormone variable, that is, by comparing log_2_(fold changes) and FPKM values, we found that *BSKs* were the most frequent signals involved in responses to cold stress. Recently, brassinosteroids (BRs), steroid hormones other than the five classic plant hormones were discovered [49]. Several studies have shown that it can control photosynthesis, influence carbohydrate metabolism, defend plants from environmental affect [50], and confer resistance to biotic and abiotic stresses [51, 52]. BRs can activate the oxidase protection system in plants, thereby eliminating the excessive harmful free radicals derived from stresses, thereby improving the stress resistance of plants [53]. Brassinosteroid signal kinases (*BSKs*) are the downstream regulatory elements of BRs that bind to BRI1, which is located on the membrane and is activated as a substrate of BRI1 downstream of BR, thereby transmitting BR signals downstream, mediating the BR-induced increase in the activity of antioxidant protective enzymes [54]. It has been reported that *BSK5* plays an important role in drought tolerance [55]. RT-qPCR analysis revealed that the expression profile of *BSKs* coincided with the results obtained through RNA-seq. Furthermore, the BR content in clone 12–23 was significantly higher than that in clone 15–28 under cold stress, further indicating that the *BSK* gene plays an important role in cold tolerance in hypoploid *S. spontaneum*.

### Potential applications of Hypoploid *S. spontaneum* in sugarcane cold resistance breeding

In traditional polyploidy studies, the prevailing view is that polyploids have greater stress resistance [11]. Peer et al. [56] discussed the significance of polyploidy in evolution and noted that the concept of increased ploidy leading to a wide range of tolerance levels remains controversial. The results of our study indicated that hypoploid *S. spontaneum* plants are more resistant to cold than hyperploid plants. Liu et al. [28] showed that 2n = 64 *S. spontaneum* from Yunnan has better quality and yield than the other four *S. spontaneum* ploidies (2n = 64, 72, 80, 96). Zhang et al. [2] identified 80% of disease resistance genes on the rearranged chromosomes of haploid material (2n = 32) derived from the pollen culture of hypoploid *S. spontaneum*, indicating that the reduction in chromosome number facilitated the retention of disease resistance genes. Yu et al. [27] showed that ploidy evolution and the distribution of *S. spontaneum* may be correlated with habitat features, such as latitude and altitude. The systematic evolution of different ploidies of *S. spontaneum* and their stress tolerance thus merit further investigation.

Sugarcane breeding plays an important role in improving sugarcane yield and resistance. However, due to insufficient development and rational utilization of germplasm resources, as well as a long breeding cycle [7], sugarcane breeding is relatively slower than other crops. *S. spontaneum* is the most important wild sugarcane germplasm [57] and is a typical polyploid resource that is rich in resistance-related genes [58]. An in-depth study of the resistance of different ploidies of *S. spontaneum* and their rational use are thus of profound significance for accelerating sugarcane breeding. In our study, morphological, physiological, and transcriptomic analyses showed that the hypoploid *S. spontaneum* clone 12–23 (2n = 54) possesses strong cold tolerance and can be utilized to generate novel cold-tolerant sugarcane varieties. The results of our study improve our understanding of the morphological characteristics and molecular mechanisms of cold tolerance in hypoploid *S. spontaneum*.

## Conclusions

In this study, the cold tolerance of hypoploid and hyperploid clones of *S. spontaneum* was analysed through morphological, physiological, and transcriptomic data. Compared with the hyperploid clones 15–28, the hypoploid clones 12–23 exhibited different morphological and physiological characteristics, and exhibited significant tolerance to cold. The transcriptomic data showed that low temperature stress altered the expression of many stress response genes and pathways in clone 12–23, particularly sugar and starch metabolic pathways and the plant hormone signalling pathway. Our findings suggest that under low temperature conditions, the hypoploid *S. spontaneum* clone maintains a more robust osmotic regulatory system than the hyperploid clone through sugar accumulation and hormonal changes, and thus has a stronger resistance to low temperatures. The results of this study help to elucidate the morphological characteristics and molecular mechanisms underlying the strong cold tolerance of hypoploid *S. spontaneum*.

## Methods

### Plant materials

*S. spontaneum* cold-resistant clone 12–23 (2n = 54, collected from Tibet, China) and the cold-sensitive clone 15–28 (2n = 92, collected from Burma) were harvested from the resource conservation room of Sugarcane Research Institute, Yunnan Agricultural University in Kunming city, Yunnan Province, China. The ploidy identification of two clones is shown in Additional file 6: Figure S3.

### Morphometric measurement

At the mature stage, the agronomic traits of the clones were investigated. Five plants from each clone were selected for measuring plant height, stem diameter, leaf length, leaf width, and internode length (Additional file 7: Table S3). Assessment of traits was based on the Technical Code for Evaluating Crop Germplasm Resources—Sugarcane (NY/T1488–2007) [59].

### Microscopic observation

At maturity, three disease-free plants with the same growth status were selected from the two clones. Three leaves in the middle of each plant were collected for paraffin sectioning. We fixed the fresh tissue with fixative for more than 24 h, removed the tissue from the fixative and placed it into the dehydrator (Wuhan Junjie Electronics Co., Ltd., JJ-12 J) for dehydration with gradient alcohol. Dehydrated samples were embedded in an embedding machine (Wuhan Junjie Electronics Co., Ltd., JB-P5). The embedded paraffin sections were placed on a microtome (Shanghai Leica Instrument Co., Ltd., RM2016) for sectioning, and each section was 3 μm thick. Finally, dewaxing, safranin O–fast green staining, and photography were conducted, and the cross-sectional anatomy of the leaves was assessed by Caseviewer 2.0 software [11]. The stomata were observed using a scanning electron microscope. First, we limited mechanical damage when taking the material, quickly taking each sample within 1–3 min. The tissue block did not exceed 3 mm^2^, and was quickly placed in the electron microscope fixing solution (Servicebio, G1102) for 2 h at room temperature. Then the samples were transferred to a 4 °C refrigerator and stored in the dark. Then, the leaves were removed and dehydrated with ethanol. After dehydration, the sample was placed in a critical point dryer (Quorum, K850) for drying. Finally, the sample was placed on the double-sided adhesive tape of the conductive carbon film and placed on the sample table of the ion sputtering apparatus (IXRF, MSP-2S). The gold was sprayed on the sample stage of the ion sputtering apparatus (HITACHI, SU8010) [11].

### Physiological measurements

Seedlings (stage with 5–6 leaves) of *S. spontaneum* were subjected to cold stress in an incubator. The growth conditions were as follows: temperature, 3 °C; light intensity, 250–300 μmol·m^− 2^·s^− 1^; photoperiod, 12 h; and relative humidity 60–70%. After 3 days of cold stress treatment [5], the leaves of the seedlings from the control and low temperature stress treatments were collected, and 3 replicates of sampling were used per treatment. The + 1 leaves (top visible dewlap leaf) of the same size and phenotype were collected as the test material. Cell membrane permeability was measured as described by Huang et al. (2015) using a conductivity meter (DDSJ-308F, Leici, China) [60]. The leaf relative water content index was determined by selecting the + 1 leaf and recording its fresh weight (labelled as W_f_) according to the method described by Cia et al. [61]. Then, each leaf was soaked in water for 5–6 h, dried, and weighed. Then each leaf was soaked in water for 1 h, dried, and weighed again. This operation was repeated until the saturated weight of the sample was stable, and the saturated fresh weight was recorded (labelled W_t_). Then G2 was dried in an oven to a constant weight, and the dried sample was weighed (labelled W_d_). We calculated the relative water content according to the formula: (W_f_ − W_d_) / (W_t_ − W_d_) × 100%. Soluble sugar content was determined using the fluorenone colorimetric method [23]. The soluble protein content was determined using the Coomassie Brilliant Blue G^− 250^ method [23]. Superoxide dismutase, peroxidase, trehalose, and brassinosteroid contents were determined using a kit manufactured by Suzhou Keming Biotechnology Co., Ltd. The determination was performed in triplicate for each sample.

### RNA-seq analysis

The treatment of the plant materials was the same as that in Section 1.3, which included cold stress and the control. Clone 12–23 under cold stress was labelled LL, and the control was labelled LC. Clone 15–28 under cold stress was labelled HL, and the control was labelled HC. Three biological replicates were used for each treatment. The + 1 leaves (top visible dewlap leaf) of basically the same size and phenotype were used in RNA extraction and RNA-seq analysis. RNA quality was detected using a NanoDrop. Clean reads were assembled using Trinity software (version 2.0.6) [62]. The obtained transcriptomic sequences were submitted to the databases of NCBI non-redundant protein sequences (NR) and nucleotide sequences (NT), protein family (Pfam), Eukaryotic Orthologue Groups (KOG), Kyoto Encyclopedia of Genes and Genomes (KEGG), and Gene Ontology (GO) to perform BLASTX analysis to obtain the best annotation (E-value≤1E-03). Gene expression levels were assessed using RSEMV 1.2.12 software [63]. The expression level of a single gene was calculated using the expected number of fragments per kilobase of transcript sequence per million base pairs sequenced (FPKM) formula. Differentially expressed genes (DEGs) were defined using log_2_|Fold Change| > 1 and FDR (false discovery rate) < 0.05.

### Real-time fluorescent quantitative PCR detection

Real-time fluorescent quantitative reverse transcription PCR (RT-qPCR) was used to quantify the relative expression levels of genes. RNA of the leaf samples described above was extracted for RT-qPCR analysis. *GAPDH* [64] and *18S rRNA* [65, 66] genes were used as internal references. The RNA OD value was measured using an Analytik Jena™ Scandrop™ 100 Spectrophotometer, and the A260/A280 ratio was calculated. The TRUEscript 1st Strand cDNA synthesis kit manufactured by Aidlab was used for reverse transcription. A 20-μL reaction system (Additional file 8: Table S4) was used, and each reaction was performed thrice. After amplification, a melting curve was generated, and the 2^−ΔΔCt^ method was used to calculate the relative expression levels of genes [67]. The primers used for RT-qPCR are listed in Additional file 9: Table S5.

### Data analysis

Microsoft Excel 2010 was used in the data analysis. SPSS (v17.0, IBM, New York, NY, US) was used in variance analysis [68]. SigmaPlot (v10.0, Chicago, IL, US) software was used to contruct graphs [69]. HemI (v1.0, China) software was used to prepare heat maps (http://hemi.biocuckoo.org/index.php) [70].

## Supplementary information

**Additional file 1: Supplement**.

**Additional file 2: Table S1.** Overview of transcriptome sequencing and de novo assembly results.

**Additional file 3: Table S2.** Summary of the functional annotation of assembled unigenes.

**Additional file 4: Figure S1**. Analysis of variance of RT-qPCR expression of *TPS* and *TPP*.

**Additional file 5: Figure S2**. Analysis of variance of RT-qPCR expression of *BSK*.

**Additional file 6: Figure S3**. Ploidy identification of 12–23 clones and 15–28 clones.

**Additional file 7: Table S3.** Index and content of investigation on agronomic characters.

**Additional file 8: Table S4**. Quantitative real-time PCR.

**Additional file 9: Table S5**. Primers used in quantitative real-time RT-qPCR validation of gene expression data as revealed by RNA-seq analysis.

## Data Availability

The data supporting the conclusions of this article are within the paper and its additional files. All sequencing reads are deposited in the National Center for Biotechnology Information under the BioProject number PRJNA589716 with the Sequence Read Archive (SRA) study accession SRP230310.

## Abbreviations

DEGs: Differentially expressed genes
KEGG: Kyoto Encyclopedia of Genes and Genomes
GO: Gene Ontology
KOG: Eukaryotic Orthologue Groups
NT: Nucleotide sequences
NR: Nonredundant protein sequences
Pfam: Protein family
FPKM: Ragments per kilobase of transcript sequence per million base pairs sequenced
FDR: False discovery rate
