# Supplementary material for: Enhanced sugar accumulation and regulated plant hormone signalling genes contribute to cold tolerance in hypoploid Saccharum spontaneum
Source: BMC Genomics. 2020 Jul 22;21:507. doi: 10.1186/s12864-020-06917-z (PMC7376677; doi:10.1186/s12864-020-06917-z)
Supplement: Supplementary file 1 — Additional file 1: Supplement. [file 12864_2020_6917_MOESM1_ESM.docx]

In the previous work, based on the physiological indexes of plasma membrane permeability (PMP) and relative water (RWC), chlorophyll (CHL), soluble sugar (SS), soluble protein (SP), superoxide dismutase (SOD), peroxidase (POD), and abscisic acid (ABA) contents of these materials, low-temperature stress, analysis of variance, correlation analysis, principal component analysis, membership function analysis, cluster analysis and stepwise regression were used to comprehensively evaluate the cold tolerance of 36 *S. spontaneum* clones with different ploidy levels, the result shows clone 12-23 is the cold-resistant and clone 15-28 is the cold-sensitive. Moreover, manuscript entitled “Comprehensive evaluation of cold tolerance of Saccharum spontaneum L. with different ploidy levels based on physiological indexes after low-temperature stress”, which we have submitted it to the Journal of plant biology. So this part of the data does not publicly available.
