# Supplementary material for: Enhanced sugar accumulation and regulated plant hormone signalling genes contribute to cold tolerance in hypoploid Saccharum spontaneum
Source: BMC Genomics. 2020 Jul 22;21:507. doi: 10.1186/s12864-020-06917-z (PMC7376677; doi:10.1186/s12864-020-06917-z)
Supplement: Supplementary file 2 — Additional file 2: Table S1. Overview of transcriptome sequencing and de novo assembly results. [file 12864_2020_6917_MOESM2_ESM.docx]

**Table S1 Overview of transcriptome sequencing and de novo assemble results**

|  | **LL** | **LC** | **HL** | **HC** |
| --- | --- | --- | --- | --- |
| **Clean reads** | 55300316 | 60412261 | 58497202 | 45591750 |
| **Q_20_（%）** | 97.16 | 97.40 | 97.48 | 97.15 |
| **Q_30_（%）** | 92.48 | 92.99 | 92.96 | 93.80 |
| **GC（%）** | 50.03 | 54.84 | 55.27 | 56.17 |

**Note:** Clean reads indicates filtered sequencing data; Q_20_ and Q_30_ indicates the percentage of bases greater than 20 or 30 in the phred population; GC indicates the total number of bases G and C as a percentage of the total number of bases.
