# Supplementary material for: Enhanced sugar accumulation and regulated plant hormone signalling genes contribute to cold tolerance in hypoploid Saccharum spontaneum
Source: BMC Genomics. 2020 Jul 22;21:507. doi: 10.1186/s12864-020-06917-z (PMC7376677; doi:10.1186/s12864-020-06917-z)
Supplement: Supplementary file 3 — Additional file 3: Table S2. Summary of the functional annotation of assembled unigenes. [file 12864_2020_6917_MOESM3_ESM.docx]

**Table S2 Summary of the functional annotation of assembled unigenes**

| **Public database** | **Number of unigenes** | **Percentage(%)** |
| --- | --- | --- |
| **Annotated in NR** | 55836 | 49.25 |
| **Annotated in NT** | 75297 | 66.41 |
| **Annotated in KO** | 15236 | 13.43 |
| **Annotated in SwissProt** | 31155 | 27.48 |
| **Annotated in PFAM** | 38287 | 33.77 |
| **Annotated in GO** | 38287 | 33.77 |
| **Annotated in KOG** | 7990 | 7.04 |
| **Annotated in all Databases** | 5215 | 4.6 |
| **Annotated in at least one Database** | 86275 | 76.1 |
| **Total Unigenes** | 113367 | 100 |
