# Supplementary material for: Enhanced sugar accumulation and regulated plant hormone signalling genes contribute to cold tolerance in hypoploid Saccharum spontaneum
Source: BMC Genomics. 2020 Jul 22;21:507. doi: 10.1186/s12864-020-06917-z (PMC7376677; doi:10.1186/s12864-020-06917-z)
Supplement: Supplementary file 4 — Additional file 4: Figure S1. Analysis of variance of RT-qPCR expression of TPS and TPP. [file 12864_2020_6917_MOESM4_ESM.docx]

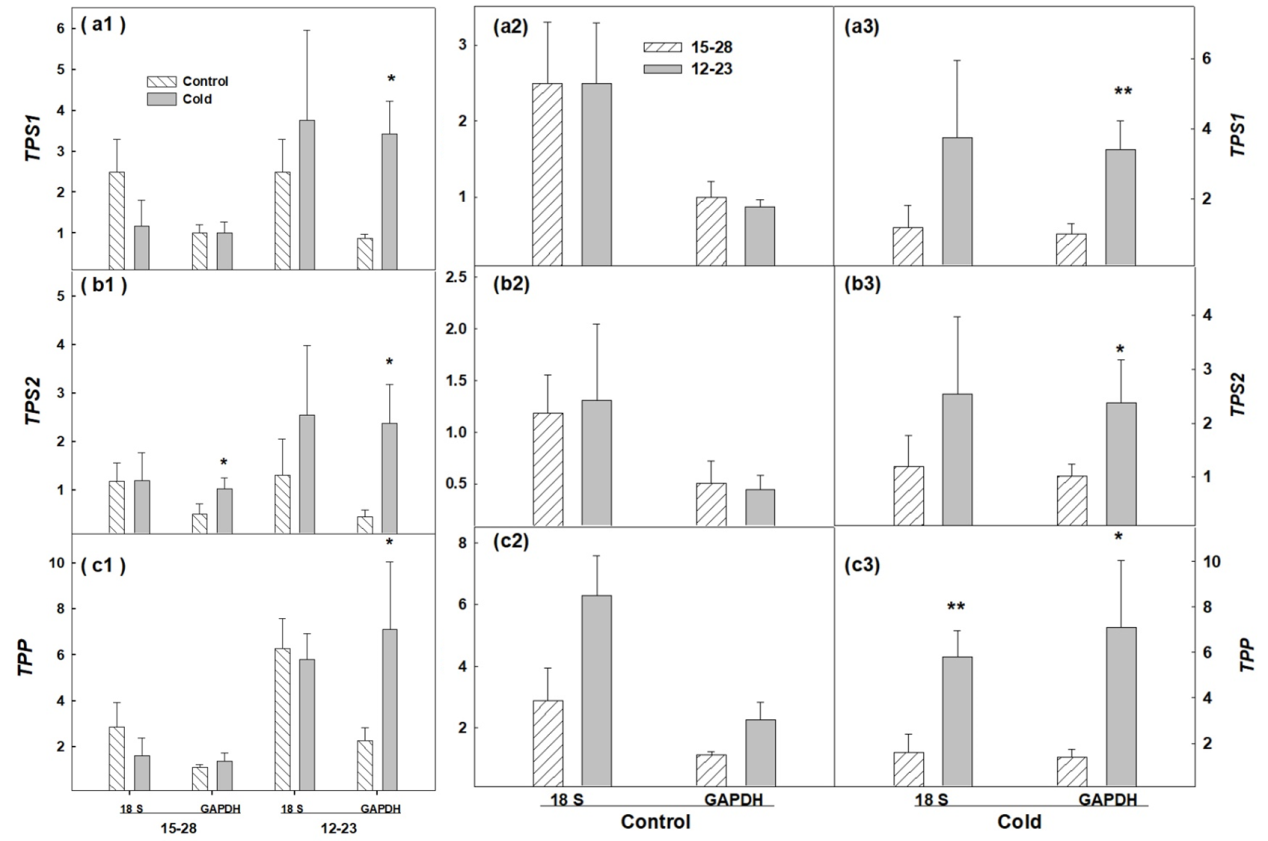


**Figure S1** Analysis of variance of RT-PCR expression of *TPS* and *TPP*. (a1)、(b1)、(c1) Analysis of variance of RT-qPCR expression levels of three genes (*TPS1, TPS2* and *TPP*) under normal temperature and low temperature stress: (a1) *TPS1*; (b1) *TPS2*; (c1) *TPP*. (a2)、(b2)、(c2) Analysis of variance in RT-qPCR expression of three genes (*TPS1*, *TPS2* and *TPP*) of clones 12-23 and 15-28 at room temperature: (a2) *TPS1*; (b2) *TPS2*; (c2) *TPP*. (a3)、(b3)、(c3) Analysis of variance in RT-qPCR expression of three genes (*TPS1*, *TPS2* and *TPP*) of clones 12-23 and 15-28 at low temperature: (a3) *TPS1*; (b3) *TPS2*; (c3) *TPP*.

The RT-qPCR expression levels of two internal reference genes of *TPS1* gene were compared by variance analysis. The results showed that RT-qPCR expression levels of *18S* and *GAPDH* were not significant for clones 15-28 under normal temperature and low temperature stress. For 12-23 clones, RT-qPCR expression of two internal reference genes under low temperature stress was higher than that under normal temperature, under normal temperature and low temperature stress, RT-qPCR expression of 18S internal reference gene was not significant, but the expression of RT-qPCR of *GAPDH* internal reference gene is significant (**Figure S1 a1**). At room temperature, RT-qPCR expression of two internal reference genes of clones 15-28 and 12-23 was not significant (**Figure S1 a2**). Under low temperature stress, the expression level of the two internal reference genes of clone 12-23 was much higher than that of clone 15-28, and the expression level of RT-qPCR of *GAPDH* internal reference genes of clone 15-28 and 12-23 was very significant (**Figure S1 a3**).

The RT-qPCR expression levels of two internal reference genes of *TPS2* gene were compared by variance analysis. The results showed that for clones 15-28, RT-qPCR expression of two internal reference genes (*18S* and *GAPDH*) under normal temperature and low temperature stress was not significant. For 12-23 clones, RT-qPCR expression of two internal reference genes was higher than that of normal temperature under low temperature stress, and RT-qPCR expression of *18S* internal reference gene was not significant under normal temperature and low temperature stress, while RT-qPCR expression of *GAPDH* internal reference gene was significant (**Figure S1 b1**). At room temperature, RT-qPCR expression of two internal reference genes of clones 15-28 and 12-23 was not significant (**Figure S1 b2**). Under low temperature stress, the expression levels of the two internal reference genes of clone 12-23 were much higher than those of clone 15-28, and the expression levels of *GAPDH* internal reference genes of clones 15-28 and 12-23 were significant (**Figure S1 b3**).

The RT-qPCR expression levels of two internal reference genes of *TPP* gene were compared by variance analysis. The results showed that RT-qPCR expression levels of *18S* and *GAPDH* were not significant for clones 15-28 under normal temperature and low temperature stress. For 12-23 clones, under normal temperature and low temperature stress, RT-qPCR expression of 18S internal reference gene was not significant, but the expression of RT-qPCR of *GAPDH* internal reference gene is significant (**Figure S1 c1**). At room temperature, RT-qPCR expression of two internal reference genes of clones 15-28 and 12-23 was not significant (**Figure S1 c2**). Under low temperature stress, the expression levels of two internal reference genes of 12-23 clones were much higher than that of 15-28 clones, among which the expression levels of RT-qPCR of the *18S* internal reference genes of 15-28 and 12-23 clones were very significant, while the expression levels of *GAPDH* internal reference genes were significant (**Figure S1 c3**).
