# Supplementary material for: Enhanced sugar accumulation and regulated plant hormone signalling genes contribute to cold tolerance in hypoploid Saccharum spontaneum
Source: BMC Genomics. 2020 Jul 22;21:507. doi: 10.1186/s12864-020-06917-z (PMC7376677; doi:10.1186/s12864-020-06917-z)
Supplement: Supplementary file 5 — Additional file 5: Figure S2. Analysis of variance of RT-qPCR expression of BSK. [file 12864_2020_6917_MOESM5_ESM.docx]

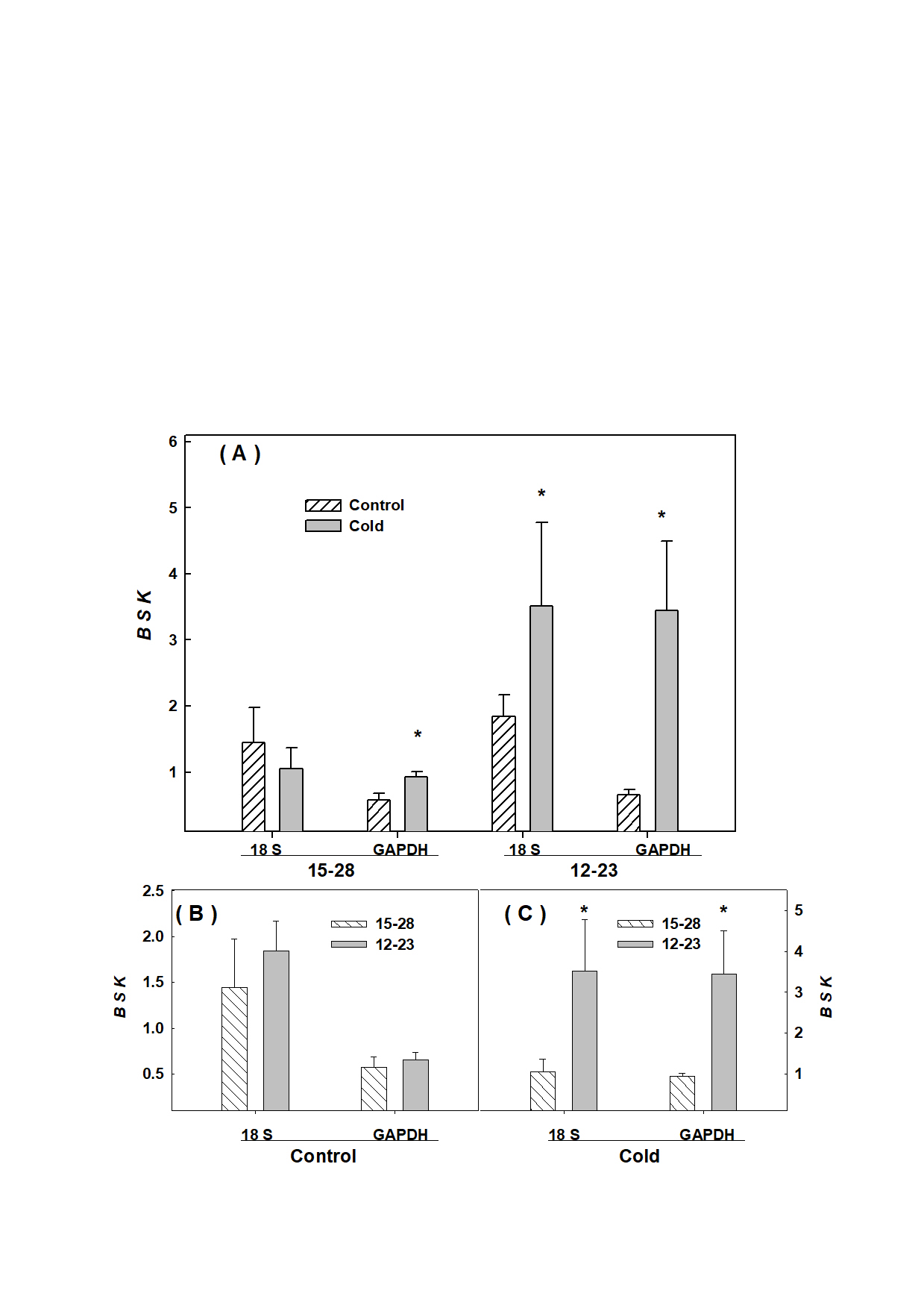


**Figure S2** Analysis of variance of RT-PCR expression of *BSK*. (A) Analysis of variance of RT-qPCR expression levels of *BSK* under normal temperature and low temperature stress; (B) Analysis of variance in RT-qPCR expression of *BSK* of clones 12-23 and 15-28 at room temperature; (C) Analysis of variance in RT-qPCR expression of *BSK* of clones 12-23 and 15-28 at low temperature.

Analysis of variance was used to compare the gene expression of two internal reference genes of *BSK* gene detected by RT-PCR. The results show that for clones 15-28, under normal temperature and low temperature stress, the gene expression of the *18S* internal reference gene detected by RT-qPCR is not significant, while the *GAPDH* internal reference gene detected by RT-qPCR is significant. For clones 12-23, under normal temperature and low temperature stress, the gene expression levels detected by RT-qPCR of two internal reference genes of *18S* and *GAPDH* are significant. Moreover, RT-qPCR expression of two internal reference genes under low temperature stress was higher than that under normal temperature **(Fig S2 A)**.

At room temperature, RT-qPCR expression of *18S* and *GAPDH* in clones 15-28 and 12-23 was not significant **(Fig S2 B)**.

Under low temperature stress, RT-qPCR expression of *18S* and *GAPDH* of clones 15-28 and 12-23 was significant, and the expression of clones 12-23 was much higher than that of clones 15-28 **(Fig S2 C)**.
