# Supplementary figures and images for: Enhanced sugar accumulation and regulated plant hormone signalling genes contribute to cold tolerance in hypoploid Saccharum spontaneum
Source: BMC Genomics. 2020 Jul 22;21:507. doi: 10.1186/s12864-020-06917-z (PMC7376677; doi:10.1186/s12864-020-06917-z)

Figure S3 Ploidy identification of 12-23 clones and 15-28 clones


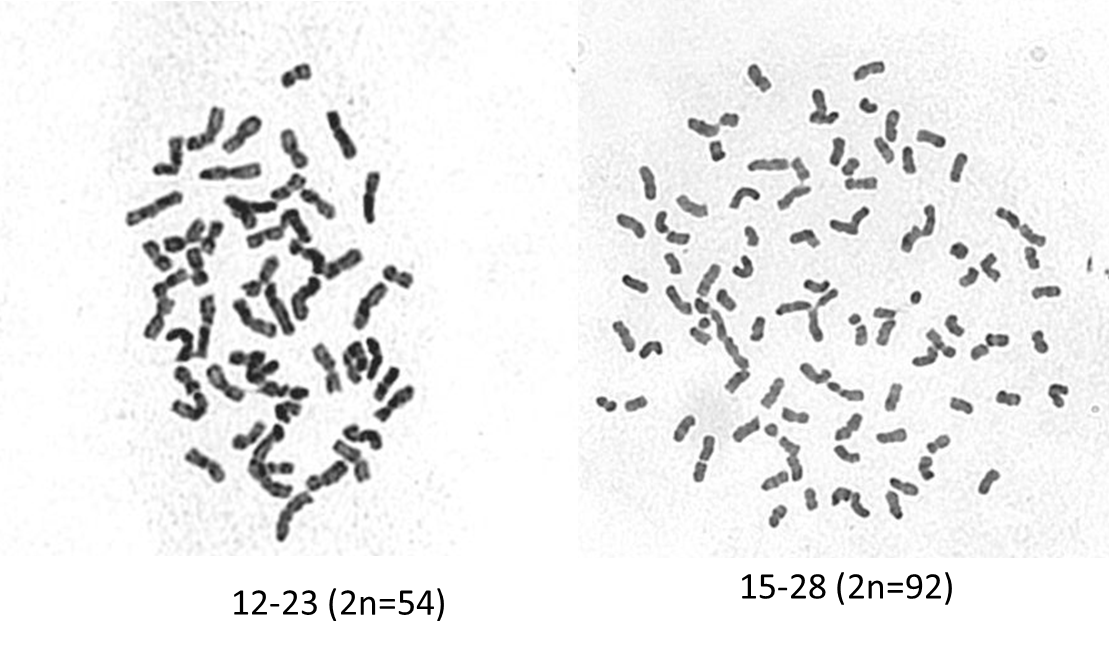

Supplement: Supplementary file 6 — Additional file 6: Figure S3. Ploidy identification of 12–23 clones and 15–28 clones. [file 12864_2020_6917_MOESM6_ESM.docx]
