# Supplementary material for: Enhanced sugar accumulation and regulated plant hormone signalling genes contribute to cold tolerance in hypoploid Saccharum spontaneum
Source: BMC Genomics. 2020 Jul 22;21:507. doi: 10.1186/s12864-020-06917-z (PMC7376677; doi:10.1186/s12864-020-06917-z)
Supplement: Supplementary file 7 — Additional file 7: Table S3. Index and content of investigation on agronomic characters. [file 12864_2020_6917_MOESM7_ESM.docx]

**Table S3 Index and content of investigation on agronomic characters**

| **Index** | **Standard of determination** |
| --- | --- |
| **Plant height** | The length of the cane stem from the ground to the highest visible hypertrophy zone was measured and the results are expressed as the mean. |
| **Stalk diameter** | The plants used for height measurement were used. The diameter of the cane stem in the middle of the plant was measured using Vernier calipers in the direction of the positive bud, and the results are expressed as the mean. |
| **Leaf length** | The length of the +3 leaf with a visible dewlap was measured form the dewlap to the tip of the leaf, and the results are expressed as the mean. |
| **Leaf width** | The length at the widest point of a leaf was measured, and the results are expressed as the mean. |
| **Internode length** | The length of the longest segment in the middle of the cane stem from the growth zone to the leaf mark was measured with a ruler and the results are expressed as the mean. |
