# Supplementary material for: Enhanced sugar accumulation and regulated plant hormone signalling genes contribute to cold tolerance in hypoploid Saccharum spontaneum
Source: BMC Genomics. 2020 Jul 22;21:507. doi: 10.1186/s12864-020-06917-z (PMC7376677; doi:10.1186/s12864-020-06917-z)
Supplement: Supplementary file 8 — Additional file 8: Table S4. Quantitative real-time PCR. [file 12864_2020_6917_MOESM8_ESM.docx]

Quantitative Real-time PCR

Reaction condition

Step1- 95 ℃-3 Min

Step2- 95 ℃-10 s

Step3- 58 ℃-30 s +plate read

Step5- Go to step2, 39 cycles

Step6- Melt curve analysis（60 ℃~ 95 ℃，+1 ℃/cycle，holding time 4 s）。

After add all components, centrifuge at 6,000rpm for 1 minute to keep all components in the bottom.

**Table S4 qPCR components and dosage**

| Components | Final concentration | Loading volume (μL) |
| --- | --- | --- |
| 2×SYBR^®^ Green Supermix | 1× | 10 |
| Reverse primer | 200nM | 1 |
| Sense primer | 200nM | 1 |
| cDNA | N/A | 2 |
| ddH_2_O | N/A | 6 |
| Total |  | 20 |
