# Supplementary material for: Enhanced sugar accumulation and regulated plant hormone signalling genes contribute to cold tolerance in hypoploid Saccharum spontaneum
Source: BMC Genomics. 2020 Jul 22;21:507. doi: 10.1186/s12864-020-06917-z (PMC7376677; doi:10.1186/s12864-020-06917-z)
Supplement: Supplementary file 9 — Additional file 9: Table S5. Primers used in quantitative real-time RT-qPCR validation of gene expression data as revealed by RNA-seq analysis. [file 12864_2020_6917_MOESM9_ESM.docx]

Table S5 Primers used in quantitative real-time RT-PCR validation of gene expression data as revealed by RNAseq analysis

| Gene ID | Primer F (5'→3') | Primer R (5'→3') | Basic annotation |
| --- | --- | --- | --- |
| **Cluster-1458.47387** | **ATACAGGCACGAACGATT** | **ATTAGCAGCAGTTACAGGAT** | **6-Phosphate glucose trehalose synthase (*TPS1*)** |
| **Cluster-1458.46702** | **GGAGGAAGCAAATAACATAATAAC** | **GACCATAGACGACAGGAG** | **6-Phosphate glucose trehalose synthase (*TPS1*)** |
| **Cluster-1458.16790** | **TACACCTCCTCCATCACC** | **TGCCTTCCAACTGACTGT** | **Trehalose phosphate phosphatase (*TPP*)** |
| **Cluster-1458.23179** | **GAATAGCAATCAATGGCAGTAG** | **CAGATGTGGACGAATCAGAT** | **Brassinosteroid signal kinase (BSK)** |
